# Unified Cox model based multifactor dimensionality reduction method for gene-gene interaction analysis of the survival phenotype

**DOI:** 10.1186/s13040-018-0189-1

**Published:** 2018-12-14

**Authors:** Seungyeoun Lee, Donghee Son, Yongkang Kim, Wenbao Yu, Taesung Park

**Affiliations:** 1 Department of Mathematics and Statistics, Sejong University, 209 Neungdong-ro, Gwangjin-gu, Seoul, 05006 South Korea; 2 Department of Statistics, Seoul National University, Shilim-dong, Kwanak-gu, Seoul, 151-742 South Korea; 3 Division of Oncology and Centre for Childhood Cancer Research, Children’s Hospital of Philadelphia, Philadelphia, PA 19104 USA

**Keywords:** Survival time, Cox model, Multifactor dimensionality reduction method, Gene-gene interaction, Unified model based method

## Abstract

**Background:**

One strategy for addressing missing heritability in genome-wide association study is gene-gene interaction analysis, which, unlike a single gene approach, involves high-dimensionality. The multifactor dimensionality reduction method (MDR) has been widely applied to reduce multi-levels of genotypes into high or low risk groups. The Cox-MDR method has been proposed to detect gene-gene interactions associated with the survival phenotype by using the martingale residuals from a Cox model. However, this method requires a cross-validation procedure to find the best SNP pair among all possible pairs and the permutation procedure should be followed for the significance of gene-gene interactions. Recently, the unified model based multifactor dimensionality reduction method (UM-MDR) has been proposed to unify the significance testing with the MDR algorithm within the regression model framework, in which neither cross-validation nor permutation testing are needed. In this paper, we proposed a simple approach, called Cox UM-MDR, which combines Cox-MDR with the key procedure of UM-MDR to identify gene-gene interactions associated with the survival phenotype.

**Results:**

The simulation study was performed to compare Cox UM-MDR with Cox-MDR with and without the marginal effects of SNPs. We found that Cox UM-MDR has similar power to Cox-MDR without marginal effects, whereas it outperforms Cox-MDR with marginal effects and more robust to heavy censoring. We also applied Cox UM-MDR to a dataset of leukemia patients and detected gene-gene interactions with regard to the survival time.

**Conclusion:**

Cox UM-MDR is easily implemented by combining Cox-MDR with UM-MDR to detect the significant gene-gene interactions associated with the survival time without cross-validation and permutation testing. The simulation results are shown to demonstrate the utility of the proposed method, which achieves at least the same power as Cox-MDR in most scenarios, and outperforms Cox-MDR when some SNPs having only marginal effects might mask the detection of the causal epistasis.

## Background

Many statistical methods in genome-wide association studies (GWAS) have been developed to identify susceptibility genes by considering a single SNP at a time. Since the first published GWAS on age-related macular degeneration [1], the GWAS Catalog has come to contain 60,000 unique SNP-trait associations based on 3300 publications as of February of 2018 (www.ebi.ac.uk/gwas). However, the effective sizes of the loci identified via GWAS are relatively small, and these individual loci may not be useful in assessing risk in personal genetics, as pointed out by Moore and Williams [2] and Manolio [3]. Furthermore, only a small proportion of heritability has been explained, leading to the missing heritability problem [4].

In order to overcome the missing heritability, the single-locus approach has been moved into gene-gene interaction analysis because complex diseases might be associated with multiple genes and their interactions [3]. However, the study of gene-gene interactions in GWAS involves the challenge of higher-order dimensionality, which Ritchie et al. [5] proposed circumventing using the multifactor dimensionality reduction (MDR) method, now commonly used to analyze gene-gene interactions in genetic studies [6, 7]. MDR reduces multi-dimensional genotypes into one-dimensional binary attributes, in which multi-level genotypes of SNPs are classified into either high or low risk groups, using a ratio of cases and controls. The MDR algorithm then finds the best pair of SNPs among all possible SNP combination, yielding the maximum balanced accuracy through cross-validation. The MDR mechanism can apply higher-order interactions such as two-way, three-way and so forth because all combinations of multi-way interactions can be reduced to either high or low risk groups using the appropriate classification rules. Many modifications and extensions to MDR have been published by generalizing the classification rules and phenotypes, including the use of odds ratios [8], log-linear models [9], a generalized multifactor dimensionality reduction method (GMDR) for generalized linear models [10], methods for imbalanced data [11], model-based multifactor dimensionality reduction methods (MB-MDR) [12] and quantitative multifactor dimensionality reduction (QMDR) for the continuous response variables [13].

On the other hand, for a prospective cohort study, the MDR concept has been also extended to investigate those gene-gene interactions associated with the survival time. Since the first approach, called Surv-MDR, was proposed by Gui et al. [14], both Cox-MDR [15] and AFT-MDR [16] have been developed for the survival phenotype. These methods extend the MDR algorithm to the survival time by using alternative classification rules, which are more applicable to survival data. For example, the classification rule for Surv-MDR corresponds to a log-rank test statistic whereas those for Cox-MDR and AFT-MDR correspond to a martingale residual of a Cox model and a standardized residual of an accelerated failure time (AFT) model, respectively. In addition, comparing the performance of these three methods, both Cox-MDR and AFT-MDR have greater power in identifying gene-gene interactions than Surv-MDR when there is a confounding covariate, whose confounding effect can be adjusted for under Cox-MDR and AFT-MDR in the frame of regression model. Whereas Surv-MDR is nonparametric and no covariate effect can be adjusted for [17].

However, the MDR algorithm requires cross-validation to identify the best multi-locus model among all possible combinations of SNPs and further implements computationally intensive permutation testing to check the significance of the selected multi-locus model. A variety of classification rules has been proposed but the intensive computational procedure for cross-validation and permutation testing should be implemented as done in the original MDR method.

Recently, the UM-MDR method has been proposed to address this issue by unifying the significance test with the MDR algorithm using regression model [17]. UM-MDR provides the significance test for the multi-locus model by introducing an indicator variable for the high risk after classification. It also allows a variety of classification rules and phenotypes.

In this paper, we proposed a simple approach, called Cox UM-MDR, which combines Cox-MDR with UM-MDR. We compared it with Cox-MDR by simulation studies. We also applied the proposed method to a real dataset of Korean leukemia patients and concluded with a discussion.

## Methods

As described in Yu et al. [18], the UM-MDR method was proposed to avoid the intensive computing procedure for achieving the significance of a multi-locus model. To this, they proposed a two-step unified model based MDR approach, in which multi-genetic levels were classified into high and low risk groups and an indicator variable for high risk group was defined in the first step, and then the significance of multi-locus model was achieved in the regression model with an indicator variable as well as adjusting covariates in the second step. The key idea of UM-MDR is to unify the algorithm of MDR and the significance testing of multi-locus model by using an indicator variable for high risk group. UM-MDR allows different types of traits and evaluation of the significance of existing MDR methods.

In this paper, we extended UM-MDR to the survival phenotype using Cox-MDR. In the first step of Cox UM-MDR, we classify the multi-level genotypes into high or low risk groups by using the martingale residual of a Cox model with only the baseline hazard function. We then define an indicator variable, *S*, taking 1 for the high-risk group and 0 for the low-risk group. In the second step, we fit a Cox model given as follows:

$$ \lambda \left(t|S,Z\right)={\lambda}_0(t)\exp \left(\beta S+{\gamma}^{\prime }Z\right) $$

Here *λ*_0_(t) is a baseline hazard function, *S* is an indicator variable for the high-risk group and *Z* is the vector coding for the adjusting covariates, *β* and γ are the corresponding parameters to *S* and *Z*, respectively. By testing the null hypothesis of *H*_0_ : *β* = 0**,** we investigate whether the corresponding multi-locus is associated with the survival time after adjusting for covariate effects. In order to test the significance of multi-locus model, we used the Wald-type test statistic, $W={\widehat{\beta}}^2/V\widehat{a}r\left(\widehat{\beta}\right)$, whose asymptotic distribution is not the central chi-square distribution under the null hypothesis [12]. This is because the expected value of the estimate of *β* is not equal to zero under the null hypothesis, which is owing to the fact that *S* represents a high-risk group classified by the martingale residual in the first step. In other words, the asymptotic null distribution of $\sqrt{W}$ has a nonzero mean due to the classification step and the asymptotic distribution of its squared statistic, *W*, follows the non-central chi-square distribution with one degree of freedom and the non-centrality parameter *q*. Since the mean of the non-central chi-square distribution is *q* + 1, we can estimate the non-centrality parameter as, $\widehat{q}=\max \left(0,\widehat{\mu}-1\right)$ where $\widehat{\mu}$ is the estimator for the mean of *W* under the null distribution. In order to estimate *q*, we permuted the trait a few times, say 5 times, and took the sample mean for statistic *W* as $\widehat{\mu}$. We can estimate the non-centrality parameter for each multi-locus model or pool all the statistics and then estimate the common non-centrality parameter for all multi-loci models as mentioned in [18].

Through intensive simulation studies, we compared the performance of Cox UM-MDR with that of Cox-MDR without and with adjusting for marginal effects. We considered two disease-causal SNPs among 10 unlinked diallelic loci with the assumption of Hardy-Weinberg equilibrium and linkage equilibrium. For the covariate adjustment, we consider only the one covariate which is associated with the survival time but has no interactions with any SNPs. We generated simulation datasets from different penetrance functions [11], which define a probabilistic relationship between the high or low risk status of groups and SNPs. We then considered 14 different combinations of two different minor allele frequencies of (0.2, 0.4) and seven different heritability of (0.01, 0.025, 0.05, 0.1, 0.2, 0.3, 0.4). For each of 14 heritability-allele frequency combinations, a total of five models were generated, yielding 70 epistasis models with various penetrance functions, as described in [11] (supplemental Table 1).

Let *f*_*ik*_ be an element from the *i*^*th*^ row and the *k*^*th*^ column of a penetrance function. Assuming that SNP1 and SNP2 are the two disease-causal SNPs, we have the following penetrance function:

$$ {f}_{ik}=P\left(\mathrm{high}\ \mathrm{risk}\ |\  SNP1=i, SNP2=k\right) $$

We generated 400 patients from each of 70 penetrance models to create one simulated dataset and repeated this procedure 100 times. We simulated the survival time from a Cox model specified as follows:

$$ \boldsymbol{\lambda} \left(\boldsymbol{t}|\boldsymbol{x},\boldsymbol{z}\right)={\boldsymbol{\lambda}}_{\mathbf{0}}\left(\boldsymbol{t}\right)\boldsymbol{\exp}\left(\boldsymbol{\alpha} \boldsymbol{x}+\boldsymbol{\gamma} \boldsymbol{z}\right) $$

Here ***x*** is an indicator variable with value 1 for the high-risk group and 0 for the low-risk group. We set ***α =*** **1.0*,γ =*** **1.0** and *z* as an adjusting covariate generated from ***N***(**0**, **1**). In addition, the baseline hazard function follows a Weibull distribution with a shape parameter of 5 and a scale parameter of 2, the censoring time being generated from a uniform distribution, *U*(0,*c*) depending on the censoring fractions which have four different censoring fractions of (0.0, 0.1, 0.3, 0.5).

For the power comparison, we consider two different scenarios for the simulation study. First, we conducted the power comparison when there is no marginal effect of SNPs. Under this scenario, the survival times are generated from the Cox model as follows:

$$ \boldsymbol{\lambda} \left(\boldsymbol{t}|\boldsymbol{x},\boldsymbol{z}\right)={\boldsymbol{\lambda}}_{\mathbf{0}}\left(\boldsymbol{t}\right)\mathbf{\exp}\left(\boldsymbol{\alpha} \boldsymbol{x}+\boldsymbol{\gamma} \boldsymbol{z}\right) $$

where ***α*** = **1.0**, ***γ*** = **1.0**.

Secondly, we compared the power of Cox UM-MDR with that of Cox-MDR when there is a marginal effect of SNPs. Under this scenario, the survival times are generated from the given Cox model as follows:

$$ \boldsymbol{\lambda} \left(\boldsymbol{t}|\boldsymbol{x},\boldsymbol{z},\boldsymbol{SNP}\mathbf{3}\right)={\boldsymbol{\lambda}}_{\mathbf{0}}\left(\boldsymbol{t}\right)\mathbf{\exp}\left(\boldsymbol{\alpha} \boldsymbol{x}+\boldsymbol{\gamma} \boldsymbol{z}+\boldsymbol{\delta} \boldsymbol{SNP}\mathbf{3}\right) $$

where ***α =*** **1.0*,γ =*** **1.0*,δ =*** **0.5**, and ***δ*** denotes the marginal main effect of *SNP3* on the hazard rate.

## Results

### Simulation results

**Fig. 1:**
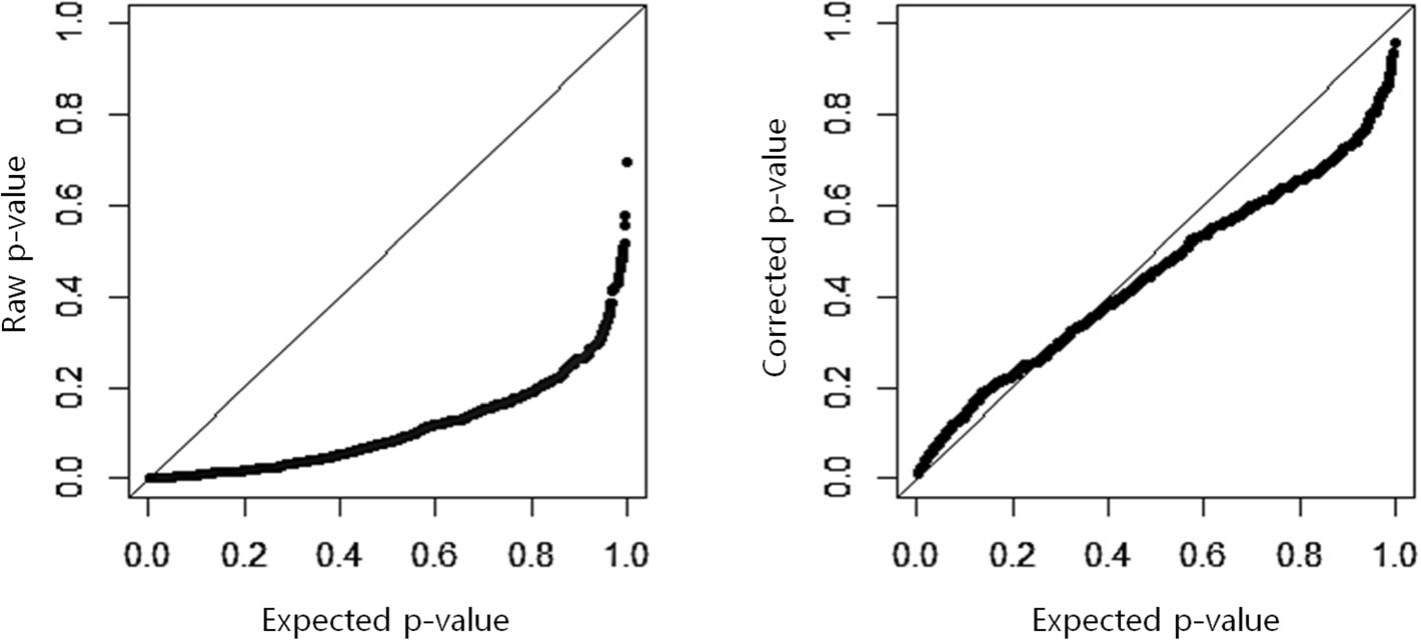
Q-Q plots for Raw and Corrected type I errors

We first considered whether the type I error is controlled under the null hypothesis. For type I error, the simulation data sets were iteratively generated 1000 times under the null hypothesis of no genetic effect model across 5 different MAFs and 4 different censoring fractions. The raw type I error was calculated without adjusting the non-centrality of the asymptotic chi-square distribution while the corrected type I error was calculated by adjusting the non-centrality. As shown in Table 1, the raw type I error is not controlled and increases as the minor allele frequency increases while the corrected type I error is well-controlled regardless of MAF. Here, we tried 5 times permutation for estimating the non-centrality because the number of permutations did not affect the test statistics, ***W*.** In addition, Fig. 1 displays Q-Q plots for the uncorrected (raw) and corrected type I errors, respectively.

**Table 1:** Raw and Corrected Type I error rates for *PBonf*

MAF	Cf = 0.0		Cf = 0.1		Cf = 0.3		Cf = 0.5	
	Raw	Corr	Raw	Corr	Raw	Corr	Raw	Corr
0.05	0.171	0.048	0.168	0.059	0.166	0.049	0.148	0.038
0.10	0.233	0.031	0.229	0.038	0.229	0.043	0.227	0.037
0.20	0.410	0.025	0.389	0.026	0.388	0.019	0.379	0.017
0.30	0.535	0.020	0.552	0.027	0.539	0.026	0.536	0.029
0.40	0.641	0.023	0.651	0.028	0.652	0.032	0.635	0.028

For the power comparison, 100 simulated datasets for each of the 70 models were generated including two disease-causal SNPs. The power of Cox UM-MDR is defined as the percentage of times that the corrected (after Bonferroni correction) *p*-value for testing the significance of the indicator variable *S* is less than or equal to the nominal size, called *PBonf,* as referred in [18]. On the other hand, the power of Cox-MDR is defined as the percentage of times that Cox-MDR correctly chooses the two disease-causal SNPs as the best model out of each set of 100 datasets for each model. This is because the significance of the best pair of SNPs selected by Cox-MDR can only be obtained by permutation testing. Therefore, the power of Cox-MDR may not be comparable with *PBonf* in terms of the evaluation measure. For a fair comparison, the alternative power of Cox UM-MDR is defined similarly as that of Cox-MDR, being the percentage of times that the causal model is ranked first by the corrected *p*-value, called *PRank*, as referred in [18]. We compared the *PBonf* and *PRank* of Cox UM-MDR with the power of Cox-MDR.

As mentioned in the previous section, we considered two different scenarios, with and without the marginal SNP effects.

Under the first scenario in which no marginal SNP effect is considered, we classified the multi-genetic genotypes into high and low risk groups using this martingale residual of a Cox model with only the baseline hazard function and define ***S*** as 1 for a high-risk group and 0 otherwise. Next, we fit the following Cox model:

$$ \lambda \left(t|S,z\right)={\lambda}_0(t)\exp \left(\beta S+\gamma z\right) $$

**Fig. 2:**
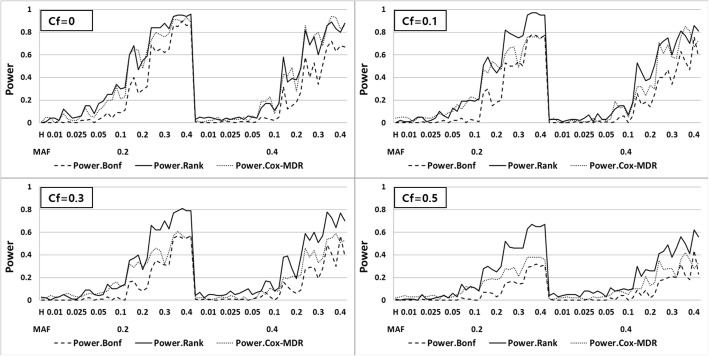
Power curves of *PRank* and *PBonf* for Cox UM-MDR and Cox-MDR without marginal effect model across the combinations of MAF, heritability and censoring fraction

In the model above, we tested the null hypothesis ***H***_**0**_ ***: β =*** **0** which means that there is no significant multi-locus effect associated with the survival phenotype. If this null hypothesis is rejected, it implies that there is a significant gene-gene interaction associated with the survival time. We overlaid the three different power curves related to Cox UM-MDR and Cox-MDR as shown in Fig. 2, in which the x-axis represents 70 models ordered by the values of 2 different MAF and 7 different heritabilities. Since there are 5 models available for each combination of MAF and heritability, a total of 70 different powers are plotted consecutively on the x-axis, in which 14 different points represents the heritability within each MAF. The power results show a consistent trend in that *PRank* of Cox UM-MDR is always greater than *PBonf* of Cox UM-MDR and the power of Cox-MDR. Under no censoring, the *PRank* of Cox UM-MDR is similar to the power of Cox-MDR but the *PRank* of Cox UM-MDR is greater than the power of Cox-MDR as the censoring fraction increases. In general, the power trend is consistent in the sense that it is more powerful for MAF = 0.2 than MAF = 0.4. The power increases as the heritability increases but decreases as the censoring fraction increases. However, the *PRank* of Cox UM-MDR seems robust even under heavier censoring than 0.5 whereas the power of Cox-MDR decreases rapidly when the censoring fraction is heavier than 0.5.

**Fig. 3:**
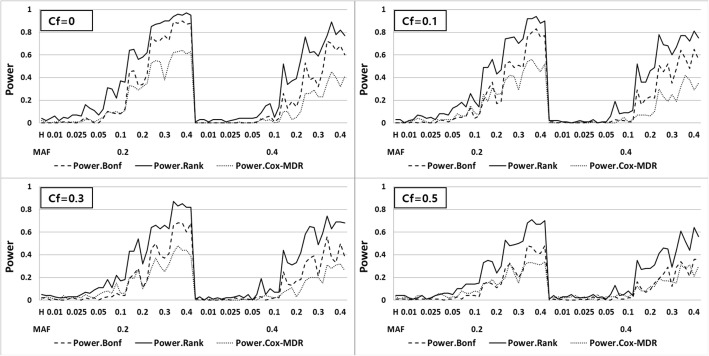
Power curves of *PRank* and *PBonf* for Cox UM-MDR and Cox-MDR with marginal effect model across the combinations of MAF, heritability and censoring fraction

Under the second scenario which marginal SNP effect is considered, we classified the multi-genetic genotypes into high and low risk groups using this martingale residual of a Cox model with only the baseline hazard function and define ***S*** as 1 for a high-risk group and 0 otherwise. Next, we fitted the following Cox model:

$$ \lambda \left(t|S,z\right)={\lambda}_0(t)\exp \left(\beta S+\gamma z+{\omega}_1 SNP1+{\omega}_2 SNP2\right) $$

where *SNP1* and *SNP2* represent the main effects of SNPs attributed to the definition of *S*. In the model above, we tested the null hypothesis of *H*_0_ : *β* = 0**,** which means that there is no significant multi-locus effect associated with the survival phenotype. Figure 3 displays the three different power curves overlaid. As shown in Fig. 3, the *PRank* of Cox UM-MDR is always largest and the *PBonf* of Cox UM-MDR is rank second in relation to the power of Cox-MDR. The general trend of these three power curves is the same as that without considering the marginal effect in terms of MAF, heritability and the censoring fraction. However, it is noted that the power of Cox-MDR is very low for almost all cases, which implies that the two-way interaction effect between SNPs can hardly be discriminated from the main marginal effect. On the other hand, Cox UM-MDR can detect the two-way interaction effect in the unified model by controlling the main effects of SNPs. As shown in Figs. 2 and 3, the *PRank* of Cox UM-MDR has reasonable power when the heritability is larger than 0.2 and seems to be robust to the censoring fraction regardless of considering the main effect of SNPs. In addition, when we compared the CPU time for the power calculation, Cox UM-MDR takes 146 s for fitting one model whereas Cox-MDR takes 1600 s, which implies that Cox UM-MDR is almost 10 times faster than Cox-MDR.

### Real data analysis

We applied the Cox UM-MDR procedure to analyze real leukemia patient data and compared the results with those obtained by Cox-MDR. This real dataset of 97 AML patients who had been followed-up which have age, sex and genetic information of 139 SNPs. At the end of the study, there were 40 deaths and 57 patients still alive. We considered two adjusting covariates, age and sex, in detecting gene-gene interaction associated with the survival time.

To take into account the marginal effect of SNP, we first fitted a univariate Cox model with each SNP adjusting for age and sex. We found that 21 SNPs had a significant marginal effect on the survival time. To summarize the marginal effects of 21 SNPs, we implemented the principal component analysis and took the two principal components (PC) as a covariate, which account for 78% of variation. We considered the four different models in identifying gene-gene interactions by Cox UM-MDR as follows:

1. PC unadjusted and main effects of SNP1 and SNP2 unadjusted:

$$ \boldsymbol{\lambda} \left(\boldsymbol{t}|\boldsymbol{S},\boldsymbol{age},\boldsymbol{sex}\right)={\boldsymbol{\lambda}}_{\mathbf{0}}\left(\boldsymbol{t}\right)\boldsymbol{\exp}\left(\boldsymbol{\beta} \boldsymbol{S}+{\boldsymbol{\gamma}}_{\mathbf{1}}\boldsymbol{age}+{\boldsymbol{\gamma}}_{\mathbf{2}}\boldsymbol{sex}\right) $$

(2) PC adjusted and main effects of SNP1 and SNP2 unadjusted:

$$ \boldsymbol{\lambda} \left(\boldsymbol{t}|\boldsymbol{S},\boldsymbol{age},\boldsymbol{sex},\boldsymbol{PC}\right)={\boldsymbol{\lambda}}_{\mathbf{0}}\left(\boldsymbol{t}\right)\mathbf{\exp}\left(\boldsymbol{\beta} \boldsymbol{S}+{\boldsymbol{\gamma}}_{\mathbf{1}}\boldsymbol{age}+{\boldsymbol{\gamma}}_{\mathbf{2}}\boldsymbol{sex}+{\boldsymbol{\delta}}_{\mathbf{1}}\boldsymbol{P}{\boldsymbol{C}}_{\mathbf{1}}+{\boldsymbol{\delta}}_{\mathbf{2}}\boldsymbol{P}{\boldsymbol{C}}_{\mathbf{2}}\right) $$

(3) PC unadjusted and main effects of SNP1 and SNP2 adjusted:

$$ \boldsymbol{\lambda} \left(\boldsymbol{t}|\boldsymbol{S},\boldsymbol{age},\boldsymbol{sex},\boldsymbol{SNP}\mathbf{1},\boldsymbol{SNP}\mathbf{2}\right)={\boldsymbol{\lambda}}_{\mathbf{0}}\left(\boldsymbol{t}\right)\mathbf{\exp}\left(\boldsymbol{\beta} \boldsymbol{S}+{\boldsymbol{\gamma}}_{\mathbf{1}}\boldsymbol{age}+{\boldsymbol{\gamma}}_{\mathbf{2}}\boldsymbol{sex}+{\boldsymbol{\theta}}_{\mathbf{1}}\boldsymbol{SNP}\mathbf{1}+{\boldsymbol{\theta}}_{\mathbf{2}}\boldsymbol{SNP}\mathbf{2}\right) $$

(4) PC adjusted and main effects of SNP1 and SNP2 adjusted:

$$ \boldsymbol{\lambda} \left(\boldsymbol{t}|\boldsymbol{S},\boldsymbol{age},\boldsymbol{sex},\boldsymbol{PC},\boldsymbol{SNP}\mathbf{1},\boldsymbol{SNP}\mathbf{2}\right)={\boldsymbol{\lambda}}_{\mathbf{0}}\left(\boldsymbol{t}\right)\mathbf{\exp}\left(\boldsymbol{\beta} \boldsymbol{S}+{\boldsymbol{\gamma}}_{\mathbf{1}}\boldsymbol{age}+{\boldsymbol{\gamma}}_{\mathbf{2}}\boldsymbol{sex}+{\boldsymbol{\delta}}_{\mathbf{1}}\boldsymbol{P}{\boldsymbol{C}}_{\mathbf{1}}+{\boldsymbol{\delta}}_{\mathbf{2}}\boldsymbol{P}{\boldsymbol{C}}_{\mathbf{2}}+{\boldsymbol{\theta}}_{\mathbf{1}}\boldsymbol{SNP}\mathbf{1}+{\boldsymbol{\theta}}_{\mathbf{2}}\boldsymbol{SNP}\mathbf{2}\right) $$

**Fig. 4:**
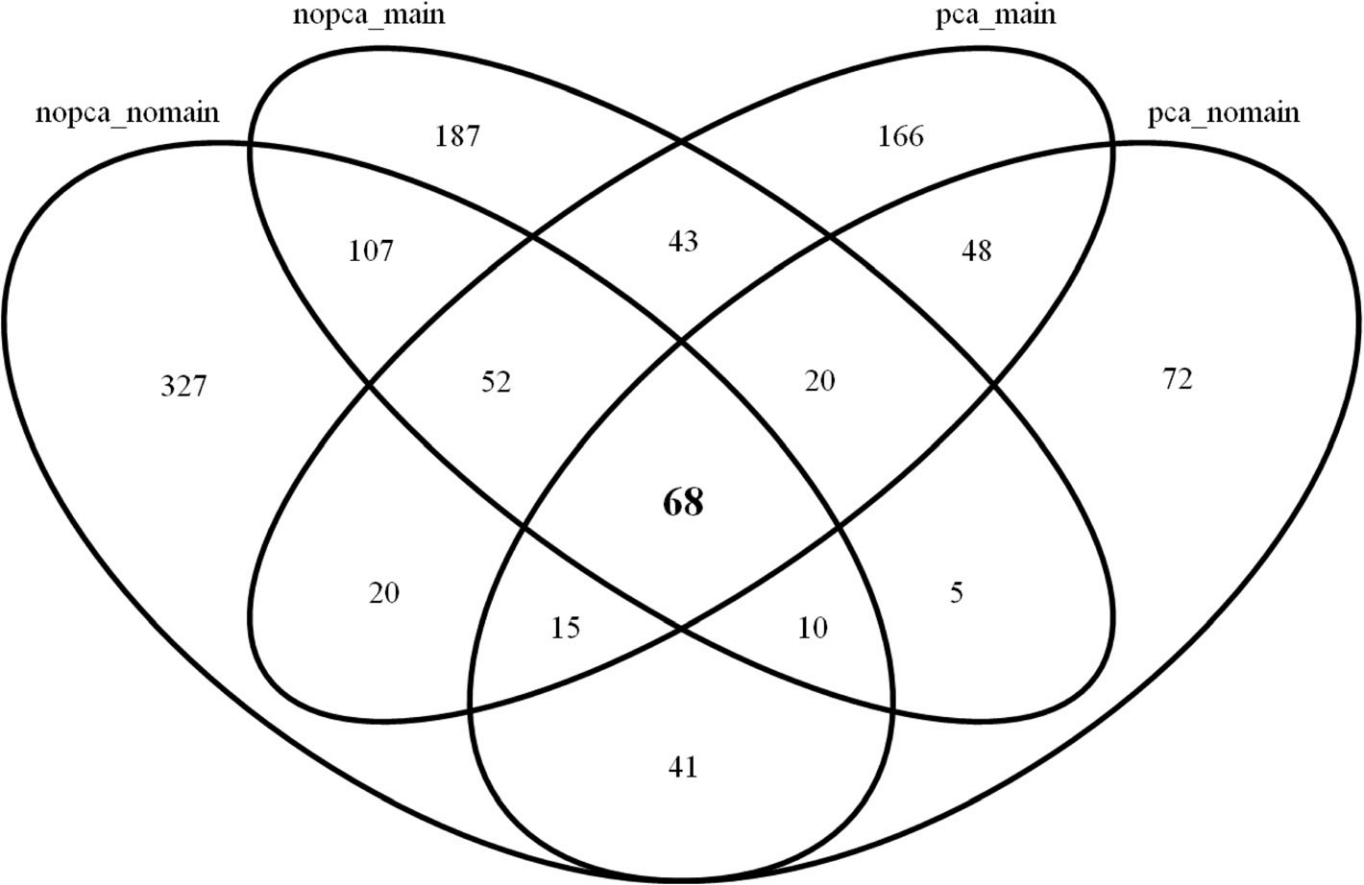
Venn diagram for the number of SNP pairs identified by the four models

The Venn diagram in Fig. 4 shows the number of SNP pairs that have a *p*-value less than 0.05 for testing *H*_0_ : *β* = 0 without adjusting multiple testing by the four models above. As shown in the Venn diagrams, 640 pairs, 279 pairs, 492 pairs and 432 pairs are detected by models (1), (2), (3) and (4), respectively. More SNP pairs are detected when the PC effect is unadjusted rather than adjusted in the model, for example, (640, 492) vs. (279, 432). The adjusting effect of PC seems more substantial when the main effect of SNPs is unadjusted since the number of SNP pairs decreases from 640 to 279. However, the adjusting effect of PC is not critical when the main effect of SNPs is adjusted because the number of SNP pairs decreases from 492 to 432. As shown in Figs. 4, 68 SNP pairs are overlapped by all four models, which imply that 68 multi-locus models might be significant with the survival phenotype regardless of the adjusting factors. For these 68 multi-locus we investigate whether the interaction effect of the corresponding SNP pairs was statistically significant or not by testing the interaction coefficient under the Cox model given as follows:

1 $$ \uplambda \left(\mathrm{t}| age, sex, SNP1, SNP2, SNP1\ast SNP2\right)={\lambda}_0(t)\exp \left({\gamma}_1 age+{\gamma}_2 sex+{\theta}_1 SNP1+{\theta}_2 SNP2+\beta\ SNP1\times SNP2\right) $$

Among the 68 SNP pairs, only 16 pairs provided statistically significant interaction effects with a *p*-value less than 0.05, which implies that Cox UM-MDR may yield more false positive results than Cox regression model. The gene-gene interaction effect can be described in various terms, for example, using a semi-parametric model like a Cox model or a nonparametric approach like Cox UM-MDR and so forth. The more important point is that the interaction effect detected by the statistical method should be interpreted from a biological point of view. However, it is not easy to connect the statistical significance directly to the biological findings.

Among the 16 pairs, we selected the top two SNP pairs and compared these with the top two SNP pairs detected by Cox-MDR. Since the Cox model is commonly used to explain the association between risk factors and survival time, we compared the power of both Cox UM-MDR and Cox-MDR by significance testing for the interaction effects in a Cox model. Table 2 shows the *p*-values for testing the interaction effects for the selected SNP pairs by both Cox UM-MDR and Cox-MDR methods, respectively. The comparison is valid since both Cox UM-MDR and Cox-MDR share a common algorithm for classifying the multi-level genotypes into high and low risk groups. Cox UM-MDR shows greater power in detecting the epistasis between two SNPs than does Cox-MDR. As shown in Table 2, the top two pairs of (rs747199, rs2847153) and (rs1960207, rs1004474) selected from Cox UM-MDR have significant interaction effects (*p* = 0.008 and 0.005), respectively. On the other hand, the top two pairs of (rs12404655, rs1004474) and (rs532545, rs2847153) show no significant interaction effect (*p* = 0.098 and 0.591), respectively. It is interesting to note that both (rs2847153) and (rs1004474) are selected simultaneously as one part of a SNP pair by both Cox UM-MDR and Cox-MDR but the interaction effect for the corresponding SNP pairs is determined by the other part of SNP pair such as (rs747199) and (rs1960207) by Cox UM-MDR. Although Cox-MDR selects the two pairs of (rs12404655, rs1004474) and (rs532545, rs2847153) as the best, the interaction effect of these pairs is not found to be significant in the Cox regression model. This is one of drawbacks of Cox-MDR method, in which it cannot be guaranteed that the best SNP pairs are statistically significant without permutation testing.

**Table 2:** Significance test for the interaction effects of top two SNP pairs identified by Cox UM-MDR and Cox-MDR

Method	SNP1	SNP2	*P*-value
Cox UM-MDR	rs747199	rs2847153	0.008
	rs1960207	rs1004474	0.005
Cox-MDR	rs532545	rs2847153	0.591
	rs12404655	rs1004474	0.098

**Fig. 5:**
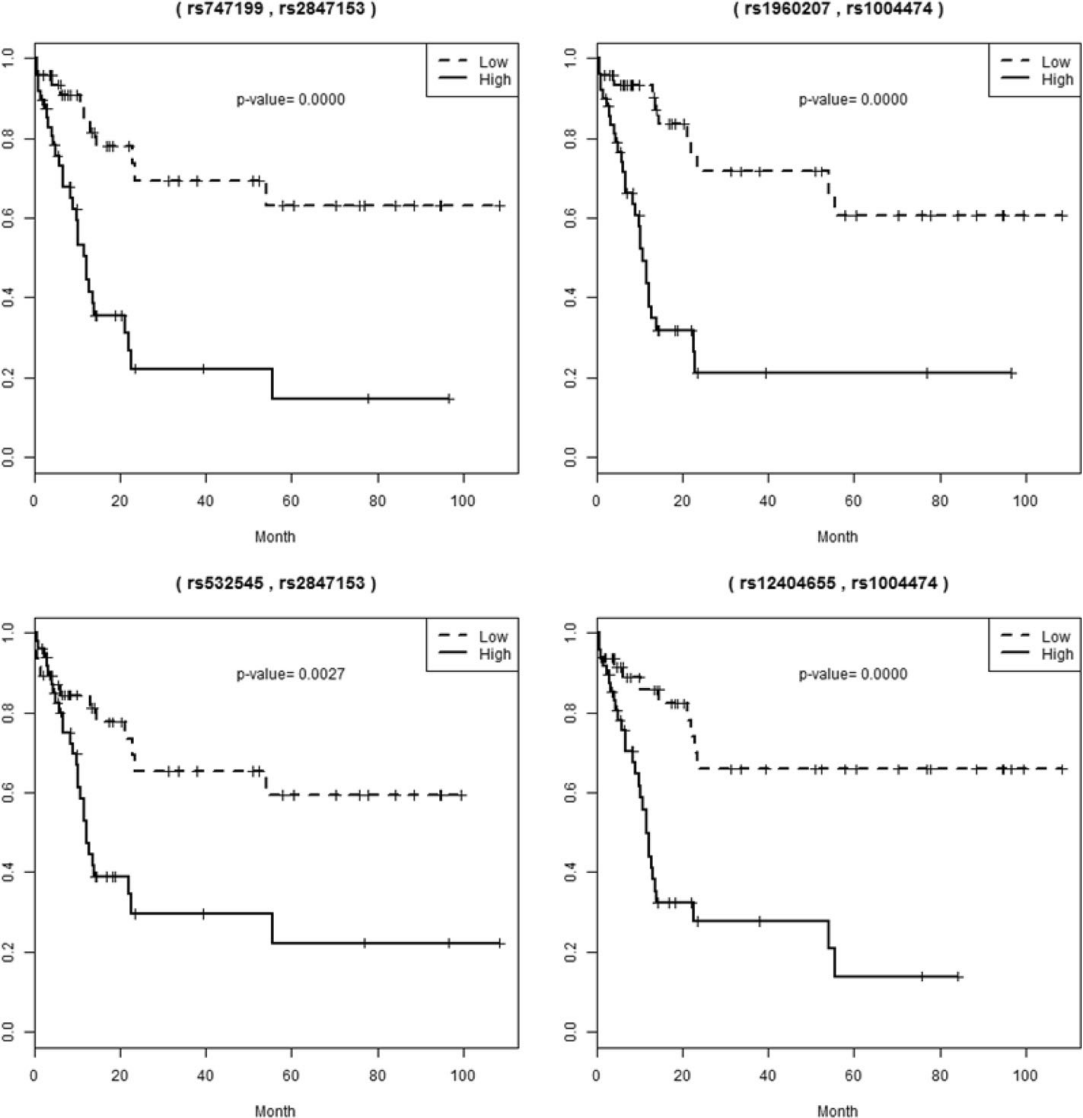
Kaplan-Meier curves for the high-risk and low-risk groups attributed by SNP pairs from Cox UM-MDR (above) and Cox-MDR (below)

In addition, we investigated how well the high and low risk groups can be classified by the SNP pairs attributed by Cox UM-MDR. To this end, we fitted a Cox model given in (1) with the attributed SNP pairs and calculated a risk score from the fitted model. We then classified all subjects into high and low risk groups based on the median risk score and tested the equivalence of the survival curves of these two groups by a log-rank test. We found significant log-rank test results with very low *p*-values for all 68 SNP pairs. Figure 5 displays four plots which include the survival curves of high-risk and low-risk groups attributed by (rs747199, rs2847153), (rs1960207 and rs1004474), (rs12404655, rs1004474) and (rs532545, rs2847153), respectively. As shown in Fig. 5, the two survival curves of high and low risk groups are significantly separated, with almost zero p-values. Furthermore, we investigated the effect of SNPs on the significant separation of these two survival curves by comparing the change of the log-rank test statistics. As displayed in Table 3, the log-rank test statistic of the no SNP effect model is 12.798, which means that two survival curves are significantly separated by both age and sex. By adding the SNP pairs attributed by Cox UM-MDR, the log-rank test statistic increases to 18.341 and 20.672, respectively, which show more powerful result. However, for the SNP pairs attributed by Cox-MDR, the log-rank test does not guarantee more powerful result because one of SNP pairs yields the lower log-rank test statistic of 8.976 whereas the other case provides the log-rank test statistic of 17.278. For all 16 SNP pairs showing the significant interaction effects, which are attributed by Cox UM-MDR, the power of the log-rank test is always greater than that under the model only with age and sex (data not given here). It would be said that the multi-locus model identified by Cox UM-MDR performs better in detecting the high risk group.

**Table 3:** Comparison of the log-rank tests between no SNP effect model and SNP effect model attributed by Cox UM-MDR and Cox-MDR

Model	Covariates	Log-rank test	*p*-value
No SNP effect model	Age, Sex	12.798	0.0003
SNP effect model by Cox UM-MDR	Age, Sex, (rs747199, rs2847153)^a^	18.341	0.0000
	Age, Sex, (rs1960207, rs1004474)^a^	20.672	0.0000
SNP effect model by Cox-MDR	Age, Sex, (rs532545, rs2847153)^a^	8.976	0.0027
	Age, Sex, (rs12404655, rs1004474)^a^	17.278	0.0000

^a^ denotes the model including two main effects of SNP1 and SNP2 and their interaction effect

## Discussion

In this paper, we proposed a simple approach, called Cox UM-MDR, which combines the classification rule of Cox-MDR with the testing procedure of UM-MDR for the survival phenotype. Through the intensive simulation study, we compared the power of Cox UM-MDR with that of Cox-MDR. The simulation results show that Cox UM-MDR is more powerful than Cox-MDR and is robust to the censoring fraction. Furthermore, we applied the proposed method to a real dataset of Korean leukemia patients and compared the results with those of Cox-MDR. We found that the results from Cox UM-MDR are more consistent than those from Cox-MDR in that the interaction effect of SNP pairs identified by Cox UM-MDR is statistically significant, whereas those identified by Cox-MDR show no significant interaction effect in a Cox model. In addition, the multi-locus model identified by Cox UM-MDR improves the power in detecting the high risk group by a log-rank test.
